# Key Genes Regulating Skeletal Muscle Development and Growth in Farm Animals

**DOI:** 10.3390/ani11030835

**Published:** 2021-03-16

**Authors:** Mohammadreza Mohammadabadi, Farhad Bordbar, Just Jensen, Min Du, Wei Guo

**Affiliations:** 1 Department of Animal Science, Faculty of Agriculture, Shahid Bahonar University of Kerman, Kerman 77951, Iran; mrm@uk.ac.ir; 2 Center for Quantitative Genetics and Genomics, Aarhus University, 8210 Aarhus, Denmark; just.jensen@qgg.au.dk; 3 Washington Center for Muscle Biology, Department of Animal Sciences, Washington State University, Pullman, WA 99163, USA; min.du@wsu.edu; 4 Muscle Biology and Animal Biologics, Animal and Dairy Science, University of Wisconsin-Madison, Madison, WI 53558, USA; wguo2@wisc.edu

**Keywords:** muscle development, myogenesis, adipogenesis, candidate gene, meat yield

## Abstract

**Simple Summary:**

Skeletal muscle mass is an important economic trait, and muscle development and growth is a crucial factor to supply enough meat for human consumption. Thus, understanding (candidate) genes regulating skeletal muscle development is crucial for understanding molecular genetic regulation of muscle growth and can be benefit the meat industry toward the goal of increasing meat yields. During the past years, significant progress has been made for understanding these mechanisms, and thus, we decided to write a comprehensive review covering regulators and (candidate) genes crucial for muscle development and growth in farm animals. Detection of these genes and factors increases our understanding of muscle growth and development and is a great help for breeders to satisfy demands for meat production on a global scale.

**Abstract:**

Farm-animal species play crucial roles in satisfying demands for meat on a global scale, and they are genetically being developed to enhance the efficiency of meat production. In particular, one of the important breeders’ aims is to increase skeletal muscle growth in farm animals. The enhancement of muscle development and growth is crucial to meet consumers’ demands regarding meat quality. Fetal skeletal muscle development involves myogenesis (with myoblast proliferation, differentiation, and fusion), fibrogenesis, and adipogenesis. Typically, myogenesis is regulated by a convoluted network of intrinsic and extrinsic factors monitored by myogenic regulatory factor genes in two or three phases, as well as genes that code for kinases. Marker-assisted selection relies on candidate genes related positively or negatively to muscle development and can be a strong supplement to classical selection strategies in farm animals. This comprehensive review covers important (candidate) genes that regulate muscle development and growth in farm animals (cattle, sheep, chicken, and pig). The identification of these genes is an important step toward the goal of increasing meat yields and improves meat quality.

## 1. Introduction

As a complex and heterogeneous tissue, skeletal muscle is considered a vital organ for the muscular system [1]. This very abundant tissue in vertebrates has different but crucial metabolic actions. Lean skeletal muscle mass regulates the body’s rate of energy expenditure [2,3,4]. Theoretically, increased muscle mass and energy expenditure from muscle protein turnover can prevent obesity [5]. Moreover, skeletal muscle has the highest insulin-stimulated glucose uptake and thus maintains the insulin sensitivity of the whole body [6]. Elevated skeletal muscle development in farm animals is important because it creates tissue that meets human requirements for meat consumption. The development of fetal skeletal muscle involves primarily myogenesis, fibrogenesis, and adipogenesis [7] derived from mesenchymal stem cells (*MSCs*). Meat quality also can be enhanced by changing the *MSC* commitment from muscle to adipocyte development through the provision of more intramuscular fat. 

Skeletal muscle development is determined mainly by the proliferation and differentiation of myoblasts, which are precursors of muscle cells. Myogenic regulatory factors (MRFs) and growth promoters are crucial for muscle development in farm animals [8]. Generally, muscle growth, categorized as a heritable feature, can be modulated by fiber type position, especially with regard to metabolism and contractile speed, as well as temperature and food availability [9,10]. Skeletal muscle has a very unique ability to regenerate and remake itself in response to growth and injury, via the activation of muscle stem cells or satellite cells [11,12].

## 2. Myogenesis

Myogenesis is the complex process of skeletal muscle construction in diverse species, including farm animals. Generally, the aim of myogenesis is the formation of multinucleated myofibers with contractile activity. The time required for each developmental phase varies among species [13]. Typically, myogenesis is regulated by the intricated networking of intrinsic and extrinsic factors [1], monitored by MRF genes in different phases as well as genes that code proteins called kinases [13]. Nutrition also plays an important role in the regulation of myogenesis. Malnutrition and overnutrition during gestation abated fetal myogenesis, but only overnutrition enhanced intermuscular fat accumulation [14,15].

Prenatal muscle development is achieved by the proliferation and differentiation of myogenic factors; postnatal muscle growth is achieved by cascade actions of muscle satellite cells. The formation of muscle in farm animals is accomplished via various biological processes, including protein accretion and muscle cell proliferation [7]. Small proportions of progenitor cells in the myotome proliferate and then differentiate into myoblasts. These myoblasts withdraw from the cell cycle and begin to differentiate and fuse to each other to construct myotubes and primary myofibers [16]. Myoblasts near primary muscle fibers proliferate and fuse to form secondary muscle fibers [17]. Muscle fibers in adult animals are established primarily through secondary myogenesis.

In the late fetal stage, some myogenic cells become quiescent, leading to the formation of satellite cells. Thus, the number of myoblasts not only conditions the quantity of muscle fibers but also influences the amount of satellite cells present during postnatal growth [14]. As the number of muscle fibers does not change after birth in most cases, fetal myogenesis is critical for efficient muscle growth in farm animals [7]. Satellite cells first proliferate and then differentiate and fuse with existing muscle fibers, leading to postnatal growth in size or hypertrophy. Satellite cells in adult animal muscles remain quiescent unless activated by extrinsic stimuli (i.e., injury and exercise). Activated satellite cells repair or regenerate fibers in affected muscle. Some age-related diseases reduce the number of satellite cells leading to impaired regeneration and muscle degeneration [18].

### Effects of Transcription Factors and the Myogenic Kinome on Myogenesis

Myogenesis is controlled mainly by specific muscle-related transcription factors, including MRFs (*MYF5*, *MYOD*, myogenin, and *MRF4*), *PAX7*, and *PAX3*. These factors act as terminal influencers of signaling procedures and contribute to proper development of each stage specific transcripts. Paired-box transcription factors are expressed first in mesoderm cells, followed by the expression of *MYF5* and *MYOD* [19]. *PAX3* expression is indispensable for skeletal muscle development; it upregulates the expression of *MYOD* during skeletal myogenesis. *PAX7* keeps satellite cells quiescent and, along with the expression of *MYF5*, plays a critical role in the development of activated myoblasts [13,20]. Rehfeldt et al. [10] noted that *MYOD* and *MYF5* are essential for the formation of muscle cell types, whereas myogenin and *MRF4* are needed to stimulate differentiation and muscle fiber construction. *MYF5*, *MYOD*, and *MRF4* are typically responsible for activating quiescent muscle stem cells and stimulating the genes required for muscle stem cell proliferation [12]. Moreover, these factors are required for myoblast differentiation and fusion into myotubes. *MYOD* is needed for the differentiation of activated myoblasts; together with myogenin and myocyte enhancer factor 2 (*MEF2*), it stimulates the process of differentiation [13]. Pownall and Emerson [21] reported that *MYOD* is able to stimulate other MRFs and thereby the expression of muscle-specific proteins in avian species.

The myoblast proliferation rate at 5–20 h in in vitro cultures was found to be greater in samples from 6-month-old Angus cattle than in those from Hereford and Wagyu × Angus cattle of the same age, and the expression of *MYF5* was notably downregulated in Wagyu × Angus relative to Angus cattle [22]. The expression of *PAX3*, *MYOD*, and *MRF4* in the pectoralis major muscle was greater in low-weight-select (LWS) chickens than in high-weight-select (HWS) chickens on the hatching day, and the expression of *PAX3*, *PAX7*, *MYF5*, *MYOD1*, *MYOG*, and *MRF4* in this muscle was greater in HWS than in LWS animals at day 28 [23]. In the gastrocnemius muscle, the expression of *PAX3*, *MYF5*, *MYOD*, and *MYOG* was greater in LWS than in HWS chickens on the hatching day, and the expression of *PAX7*, *MYF5*, *MYOD1*, and *MRF4* was greater in HWS than in LWS birds at day 28 [23]. Quantitative real-time reverse-transcription polymerase chain reaction (qRT-PCR) analysis of samples from Dzhalginsky Merino sheep showed that *MYOD1* was among 17 of 48 evaluated genes with the greatest expression in the loin muscle [24]. *MYF5*, another important regulator of myogenesis, was shown to be associated with traits related to meat quality in cattle [25] and pigs [26]. Several regulatory transcription factors, such as *MYOD1*, were identified in the biceps femoris and longissimus dorsi muscles (LDMs) of purebred (IB) and Duroc-crossbred (IB × DU) pigs; *MYOD1* was expressed in two developmental stages (birth and growth) and thus can crucially impact phenotypes [27]. For this reason, it must be considered an important candidate gene for muscle growth in pigs.

**Figure 1:**
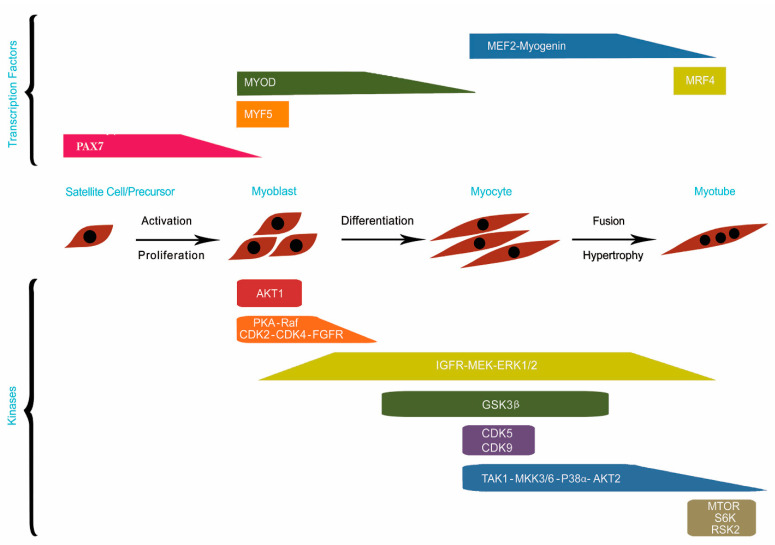
The involvement of myogenic transcription factors and protein kinases in the regulation of different stages of myogenesis. Embryonic precursors or quiescent satellite cells proliferate and construct myoblasts. Myoblasts then undergo differentiation to form myocytes. Finally, multinucleated myotubes are formed as a result of fusion of myocytes. The upper and lower sections of the figure illustrate transcription factors and protein kinases required for myogenesis, respectively (modified from [13]).

Myogenesis is not regulated solely by muscle-related transcription factors, which contribute to complex signaling cascades that precipitate the process. Protein kinases, which form a family of enzymes, modify the actions of target proteins via basic and reversible phosphorylation, a fundamental part of myogenesis. Many protein kinases have been shown to participate in different stages of myogenesis; thus, their activation or inhibition can directly affect muscle cell behavior [13]. Protein kinase A (*PKA*) is required in different muscle developmental stages and is indispensable for the construction of myogenic precursors in the dermomyotome; its involvement leads to myotome construction by myogenic factors, such as *PAX3*, *MYOD*, and *MYF5*, in dermomyotome cells [28]. This activity is initiated with the involvement of Wnt1 and Wnt7a, produced by dorsal neural tubes outside the ectoderm. The upregulation and downregulation of (Wnt)/β-catenin cascade signaling control myogenesis and adipogenesis, respectively [7]. *PKA* stimulates proliferation by promoting the expression of myogenic factors, including *MYF5*, *MYOD*, and *PAX3*, and suppressing the activity of *MEF2* via phosphorylation [13]. Cyclin-dependent kinases 2 and 4 (*CDK2*, *4*) control cell-cycle progression via the phosphorylation of retinoblastoma protein (*Rb*), which prevents *Rb* from binding the E2 factor (*E2F*), thereby allowing continued expression of cell-cycle-related genes. In addition, phosphorylated *Rb* cannot bind *MYOD*, which triggers S phase entry, allowing for the suppression of differentiation by *CDK2* and *CDK4* [29,30]. Extracellular signal-regulated kinase (*ERK*) activation depends on the availability of growth factors (*GFs*), including fibroblast growth factor (*FGF*) and insulin-like growth factor (*IGF*). In early myogenesis, *ERK1/2* activation is crucial for myoblast proliferation and the suppression of myoblast differentiation; in late myogenesis, it is required for proper myocyte fusion [13]. *Akt1* also supports proliferation by phosphorylating *FOXO1*, which suppress the expression of genes associated with cell-cycle exit, including *p27*. *p38a* has been shown to be important for the differentiation of many cell types [31,32,33]. In myogenesis, it causes myogenic precursor cells to exit the cell cycle via the phosphorylation of *MEF2* and *E47*. All of these factors together, in association with *MYOD* and phosphorylated RNA polymerase II, activated by *CDK9*, trigger differentiation. Many studies have demonstrated the role of *Akt* activity in the stimulation of differentiation and hypertrophy in culture and in vivo [34,35,36,37]. Specifically, *Akt1* seems to be crucial for myoblast proliferation but not necessary for differentiation; it inhibits differentiation when its involvement occurs alone [38,39]. Elevation of the *Akt2* level is indispensable for differentiation [38], but *Akt2* activation is not necessary for proliferation. The inactivation of *GSK3β* in response to the *Akt* level also contributes to differentiation; activated *GSK3β* suppresses myoblast differentiation and fusion [40,41,42] via the phosphorylation of *NFATC3* and *β-catenin*. *Akt* is also responsible for the maintenance of *GSK3β* inactivation via its phosphorylation during hypertrophy [42,43]. The mammalian target of rapamycin (mTOR) is required for differentiation and hypertrophy [44], but its kinase activity is just demanded for hypertrophy [45,46]. mTOR and its substrate *S6K*, whose involvement is crucial during hypertrophy, induce protein synthesis by commencing cap-dependent transfer [13]. mTOR also upregulates microRNA-1 (*miR-1*), suppressing *HDAC4* expression and upregulating important myogenic genes, such as the profusion protein follistatin [47]. Figure 1 illustrates the involvement of myogenic transcription factors and protein kinases in the regulation of different stages of myogenesis.

## 3. Adipogenesis and Participating Transcription Factors

**Figure 2:**
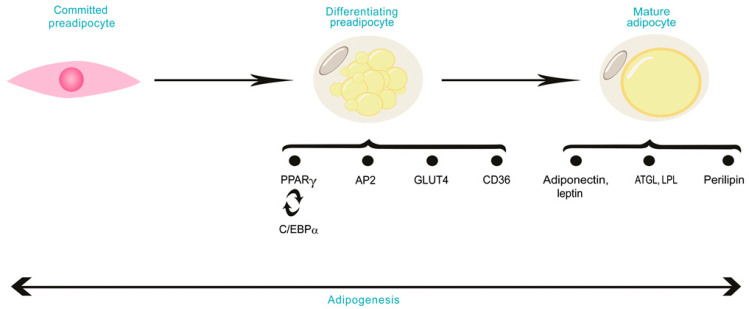
Overview of key factors involved in adipogenesis. Committed preadipocyte encounters growth arrest which leads to the formation of differentiating preadipocyte. Subsequently, differentiation occurs, and a mature adipocyte is formed.

The fundamental components of skeletal muscle are myocytes, adipocytes, and fibroblasts. Adipocytes are derived from adipogenic progenitors [48,49], produced mainly in the fetal period. The transcriptional factors that control adipogenesis include *C/EBP* and members of the *PPAR* family. *PPARα*, *γ*, and *δ/β* are nuclear hormone receptor-regulating genes associated with lipid metabolism [50]. *PPARα*, which is expressed abundantly in striated muscle, has been recognized as a mediator that stimulates peroxisomal proliferation in rodent livers [50]. Its activation enhances fatty acid β-oxidation in muscle and leads to body weight reduction [51]. Relative to *PPARα*, *PPARγ* is expressed more strongly in adipose tissues [50], stimulating adipocyte differentiation [52,53]. *PPARδ* is expressed abundantly in skeletal muscle, heart, and adipose tissue. Its active form enhances fatty acid oxidation in adipose tissue and muscle [54] and is upregulated in skeletal muscle during starvation [55]. During early adipogenesis, *C/EBPα* is stimulated and binds to the *PPARγ* promoter, stimulating its expression [56]. *C/EBPα* and *PPARγ* each induce the other’s expression in a self-reinforcing regulatory loop [7] (Figure 2) and are required for terminal adipocyte differentiation via the stimulation of genes such as adipocyte protein 2 (*AP2*), lipoprotein lipase (*LPL*), cluster of differentiation 36 (*CD36*), and glucose transporter type 4 (*GLUT4*) [57]. In bovine fetuses, myogenesis was found to be spoiled by proliferative preadipocyte-conditioned media, as demonstrated by the reduction of the fusion index, and enhanced by an adipocyte-conditioned medium, as demonstrated by the expansion of the myotube area [58]. Such crosstalk is regulated partly by adipokines, including leptin [59]. 

## 4. The Role of GFs in Skeletal Muscle Growth

Diverse types of GFs affect the differentiation and proliferation of skeletal muscle growth. Hepatocyte growth factor (*HGF*) induces the proliferation and migration of myogenic cells [60] and was found to increase the surface elasticity of bovine satellite cells in vitro [61]. *FGF2* was found to promote the proliferation of muscle precursor cells, including satellite cells and myoblasts, and to suppress cell differentiation in chickens [62]. Thus, the proper construction of muscle fibers in the embryonic period requires *FGF2* expression. However, this factor also inhibits myogenesis by suppressing *myogenin* transcription, which is required for proper myotube formation [63]. *IGFs* regulate and promote cell proliferation, differentiation, and hypertrophy and protein synthesis during myogenesis [13,64]. 

Transforming growth factor *(TGF)-β* and myostatin (*GDF-8*) have the opposite effect on differentiation [65]; thus, their expression must be limited in farm animals bred for meat. In chicken muscle, the expression of IGF-1 mRNA decreased during embryogenesis, increased after hatching, and decreased again after day 7 post-hatching, and was much greater in embryonic muscle than in embryonic liver [66]. In porcine satellite cells, *IGF-I* and *IGF-II* were upregulated during differentiation [67]. In fetal sheep, IGF-II mRNA was upregulated most at gestational day 85, demonstrating its importance during this period of differentiation and myogenic fiber formation in the leg [68]. A delay in *IGF-II* expression and mutation of the *MSTN* gene led to an increase in the number of muscle fibers in double-muscled (DM) cattle [69]. Growth hormone (*GH*) is another important factor; a major mechanism that (genetically and environmentally) affects skeletal muscle development in farm animals involves the GH–IGF axis [10].

## 5. Candidate Genes Affecting Muscle Development in Cattle

Beef production has been improved dramatically, but researchers still struggle to find quantitative trait loci (QTLs) and candidate genes affecting meat production and growth in cattle. QTLs and candidate genes related to muscle development are distributed over several bovine chromosomes (BTA), such as BTA2, 3, 4, 6, 20, and 29 [70,71,72,73,74]. Genome-wide association studies (GWASs) performed with high-density single-nucleotide polymorphism (SNP) arrays are the main means of detecting genetic variants explaining variation in such economically important traits.

Using next-generation sequencing (NGS) with nearly 12 million imputed SNPs, we identified several important candidate genes influencing hindquarter muscle development in Chinese Simmental beef cattle [74]; they included *IQUB*, *NDUFA5*, and *ASB15* in a ~322-kb area on BTA4, and *LMOD2* and *WASL* about 50 kb upstream of *ASB15*. *ASB15*, a conserved gene that is usually expressed in hypertrophied skeletal muscles [75], was found to stimulate protein turnover and synthesis, thereby inducing muscle precursor cell differentiation and muscle cell development [75,76]. *NDUFA5* upregulation has been observed in various tissues, including skeletal muscle [77], and is associated with marbling scores in Hanwoo cattle [78]. *IQUB* knockdown can suppress the expression of *c-myc*, which is involved in myoblast proliferation and differentiation [79]. *LMOD2* was found to be involved in the regulation of the length of thin filaments in muscle, and thus in muscle development in cattle [80]. *WASL* was reported to affect the cytoskeleton, a sophisticated network that plays a role in muscle contraction [81]. Based on such compelling evidence, we hypothesized that the 280 kb region in which all of these genes are located strongly influenced muscle development on BTA4 in beef cattle [74]. Using imputed NGS dataset (Illumina Hiseq 2500), we identified myotrophin (*MTPN*) as a promising candidate gene affecting net meat weight [82]. This gene stimulated six markers that regulate muscle differentiation (*MyoD*, *MyoG*, *MYH1*, *MYH2*, *MYH3*, and *MYH4*), induced myotube hypertrophy by expanding myotube diameters, and reduced myoblast proliferation in flow cytometry and cell viability experiments [82]. *MTPN* has also been shown to affect muscle development in other species, such as pig [83]. *MTPN* and *IGF-I* have been shown to induce muscle cell hypertrophy by increasing myotube diameters in chickens [84]. Using the Illumina bovine SNP50 beadchip, Kim et al. [85] identified a marker of eye muscle area (EMA) on the *DVL1* gene. Although the function of this gene in cattle is not known, research has shown that it is related to muscle development in humans and mice [86,87]. Thus, it was nominated as a candidate-gene-regulating muscle development in cattle [85]. qRT-PCR analysis showed that the expression of *NCAPG*, *LCORL*, and *DCAF16* was upregulated in the fetal relative to the adult longissimus muscle in Chinese Qichuan beef cattle, reflecting the crucial roles of these genes in early bovine muscle growth [88]. *NCAPG* was also reported to be in linkage disequilibrium with the average daily gain (ADG) in cattle [89]. Candidate genes for muscle development and growth identified in Simmental beef cattle include *MYH10* (associated with yearling weight), *RLF* (associated with ADG from birth to yearling), *SQOR* and *ARHGAP31* (associated with body weight at the age of 18 months), and *TBCB* (associated with birth weight) [90]. *TBCB* was identified as a candidate gene affecting meat quality in pigs because it is associated with actin and cytoskeleton filament development [91]. *RLF* has also been reported to markedly enhance DNA methylation of factors associated with transcriptional regulation, which is indispensable for embryonic muscle growth [92]. Mutations in the myostatin gene were found to lead to muscularity in DM cattle, causing about 20% more hypertrophy and about 50% less fat accumulation compared with normal cattle [93]. Among adipose-specific genes, *FABP4* is a promising candidate related to intermuscular fat accumulation [94]; many important differentiation-, hormone-, and fat-related regulatory factors have been identified in its promoter region. *RAI14* and *ZNF423* have also been reported to be involved in adipogenesis in bovine muscle [95,96]. cDNA microarray examination of gene expression profiles in fetal bovine longissimus muscle led to the identification of *FSTL1* and *IGFBP5* as important genes related to muscle development [97]. These genes have also been shown to be associated with *MYOD* expression in a mouse model [98].

The identification of candidate genes associated with growth is also important because these genes are potentially related to muscle development. BTA14 harbors several crucial candidate genes associated with growth, including *RPL39*, *PLAG1*, *RGS20*, *FAM110B*, *TOX*, *RP1*, *TCEA1*, *UBXN2B*, and *MRPL15* [99]. Using a high-density SNP array, Zhang et al. [100] showed that the *PLAG1–OXR1* region on BTA14 is a candidate region affecting meat yield from Wagyu cattle. *OXR1* has also been reported to regulate muscle development [101]. Its expression is regulated by the *FABP5* gene through the *PPARG* node. A GWAS led to the recognition of *RAB28*, *BTG1*, and *SMN1* as candidate genes for muscle development in Brahman cattle [102]. *RAB28* is involved in the proliferation of endothelial and smooth muscle cells [103]. *BTG1* seems to be involved in myogenic induction [104,105], and it regulates myoblast differentiation. *SMN1*, located on BTA20:8Mb, is associated with murine skeletal muscle growth [106]. Overall, the identification of candidate genes related to muscle development can be very beneficial for the beef industry, as it provides opportunities for the enhancement of meat production.

## 6. Candidate Genes Affecting Muscle Development in Sheep

An important goal of sheep breeders is to gain an understanding of the regulation of muscle growth and development, with the ultimate aim of increasing muscle growth. Recently, much research has focused on economically important traits and candidate genes influencing meat production from sheep, particularly those related to muscle development.

The most comprehensively studied QTLs in sheep are related to muscle hypertrophy. The paternal heterozygous callipyge mutation leads to skeletal muscle hypertrophy, especially in the hindquarters [107]. The callipyge variant is related to an *SNP^CLPG^* mutation in the intergenic region between *DLK1* and *GTL2* on ovine chromosome 18 (OAR18), which contains various paternally expressed genes, such as *BEGAIN* [108], *DLK1* [109], *PEG11* or *RTL1* [110], and *DIO3* [111]. It also contains several maternally expressed noncoding RNA genes, including *antiPEG11* [110], *GTL2* [112], *MIRG* [113], and *MEG8* [110]. *Dlk1* is a potent candidate gene with the potential to generate muscle hypertrophy in callipyge sheep; *Dlk1* mRNA expression was increased markedly in the muscles of these sheep from day 120 in gestation day to 84 days after birth [107]. The rib-eye muscle (Carwell) locus, which is in close proximity to *CLPG* on OAR18, has been associated with muscular hypertrophy but only of the LDM; it has no effect on carcass fatness [114]. The Carwell phenotype has been reported to enhance the loin eye area by about 11% and LDM weight by about 7% [115]. In comparison with the callipyge phenotype, the Carwell phenotype has no impact on meat quality, including meat hardness and intramuscular fat content [116]. The *MSTN* gene, which encodes myostatin, plays an important role in muscle development in sheep. In Texel sheep (known for double muscling), a mutation on *MSTN* producing a novel binding site for mRNA molecules was found to lead to muscle hypertrophy [117]; this mutation was shown to affect the meat and fat contents in lamb carcasses [118]. Gan et al. [119] identified various mutations in several regions of this gene that correlated directly with the ADG and indirectly with the double-muscling phenotype and myostatin gene function. *MSTN* is also believed to be related to calpastatin (*CAST*) expression and lamb meat quality [120,121]. In turn, *CAST* gene function is associated with protein activity in muscle and the proportion of postmortem tenderization [121,122,123]. Nikmard et al. [124] reported that *CAST* is associated with muscle mass enhancement in sheep.

**Figure 3:**
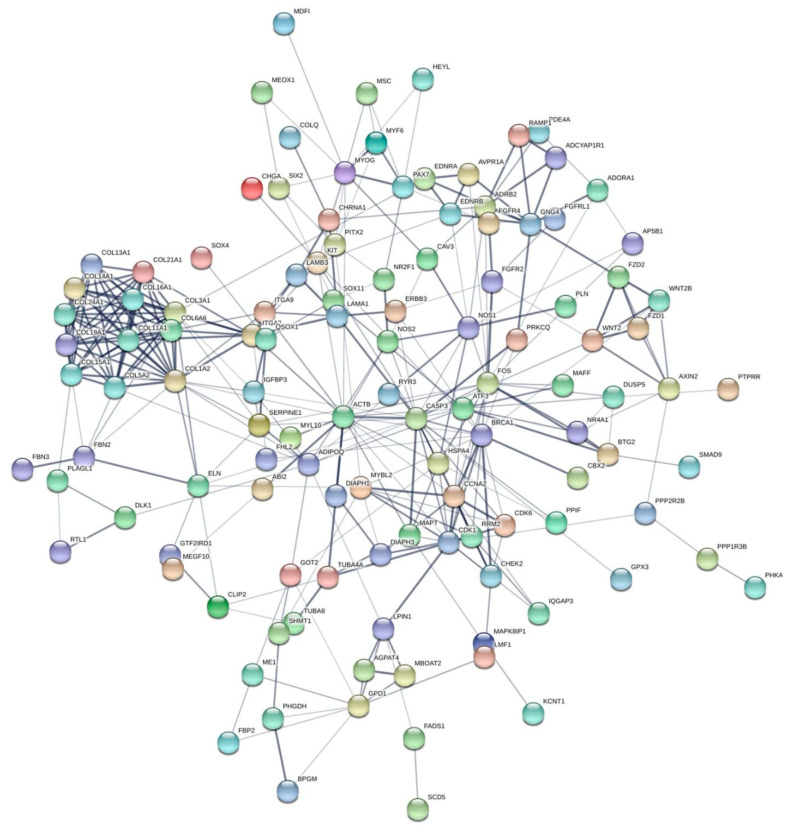
The network of differentially methylated genes associated with muscle development in sheep. Darker lines indicate higher confidence levels [131].

The *MEF2B* gene is a member of the myocyte enhancer factor-2 family, which also includes *MEF2A*, *MEF2C*, and *MEF2D*. *MEF2B* regulates muscle growth and development in sheep; PCR experiments have revealed variants in this gene that affect sheep growth and body weight [125,126]. GWASs led to the identification of several genes, including complex subunit 1 (*MSL1*), *POL*, and shisa family member 9 (*SHISA 9*), as candidate genes affecting the regulation of processes such as cell growth and muscle differentiation [125,127]. Zhang et al. [127] identified five variants in or near *MEF2B*, *RFXANK*, *RIPK2*, *GRM1*, *POL*, and *UBR2* with roles in growth and meat production. They also reported the downregulation of *TRAF6* in myoblasts, suggesting that this gene plays roles in myoblast proliferation and differentiation in sheep. Lamb carrying the *FecB* gene in a backcross population of Texel sheep exhibited a difference in meat color and greater dressing percentage and eye-muscle depth than did noncarriers [128]. Skeletal muscle transcriptome analysis revealed candidate genes affecting muscle development in Bandur sheep; genes of the Kelch superfamily (*KLHL6*, *KLHL34*, and *KLHL40*) were upregulated more than two-fold, and *ANKRD2* and *KLH13* were found to be involved in muscle development [129]. Based on analysis of gene expression profiles in ovine skeletal muscle from five Spanish meat breeds, candidate genes related to muscle contraction, including actin α1 (*ACTA1*), myosin light chain phosphorylatable fast skeletal muscle (*MYLPF*), myosin heavy chains 2 (*MYH2*) and 7 (*MYH7*), tropomyosin 2 (*TPM2*), and titin (*TTN*), were identified [130]. Fan et al. [131] employed whole-genome bisulfite sequencing to examine genome-wide DNA methylation profiles in Hu sheep and identified many important differentially methylated genes associated with muscle growth and metabolism (Figure 3). Based on network analysis, they identified nine candidate genes (*ADIPOQ*, *CCNA2*, *ITGA2*, *MYOG*, *MAPT*, *DIAPH1*, *NR4A1*, *DLK1*, and *COL1A2*) that may be associated with muscle cell proliferation and differentiation and determined that their regulation across DNA methylation can control differential muscle development. In Icelandic sheep, Guðmundsdóttir [132] identified several candidate genes for muscle growth and development (*CSF3R*, *ADAM17*, *GADD45B*, *GRID2*, *SPG11*, *DAB2*, *FREM3*, *GAB1*, *KLF13*, *AKAP6*, *PNN*, *DOCK1*, and *TRRAP*) near the most closely associated SNPs. Flicek et al. [133] also reported many genes associated with muscle growth and development and muscle fiber development in sheep [*USMG5* (Chr 13), *IFRD1* (Chr 4), *MSC* (Chr 9), *PPP2R3A* (Chr 1), *PITX1* (Chr 5), *TCF21* (Chr 8), *CACNA1S* (Chr 12), *PITX2* (Chr 6), *MYOG* (Chr 12), and *MYOD1* (Chr 15)]. A comprehensive analysis of genome-wide DNA methylation in the LDMs of Dorper × small-tailed Han and small-tailed Han sheep revealed candidate genes potentially associated with muscle production and growth (*ACOX2*, *PPARG2*, *NTN1*, *RIN2*, *MAPRE1*, *ACSL1*, *SH3PXD2B*, *ADAMTS2*, *MYOM1*, *ZDHHC13*, *TGFB3*, and *RYR1*) [134]. Various mutations in the leptin gene associated with muscle growth, meat quality, and carcass fat content have been identified in sheep [135,136]. The *FABP4* gene has an important effect on meat quality in sheep, as it does in cattle; polymorphism of this gene affects the marbling, shear force, and intramuscular fat content of the LMD [116]. In addition, *FABP4* allele A promotes lamb meat tenderness [137].

## 7. Candidate Genes Affecting Muscle Development in Pigs

Many researchers have examined pig muscle characteristics (e.g., growth and development) in efforts to improve quantity and quality of pig meat and pig growth rates. The identification of additional candidate genes would aid understanding of the mechanisms underlying pig muscle growth, benefitting breeders and consumers and contributing to the improvement of efficiency in the pork industry.

**Figure 4:**
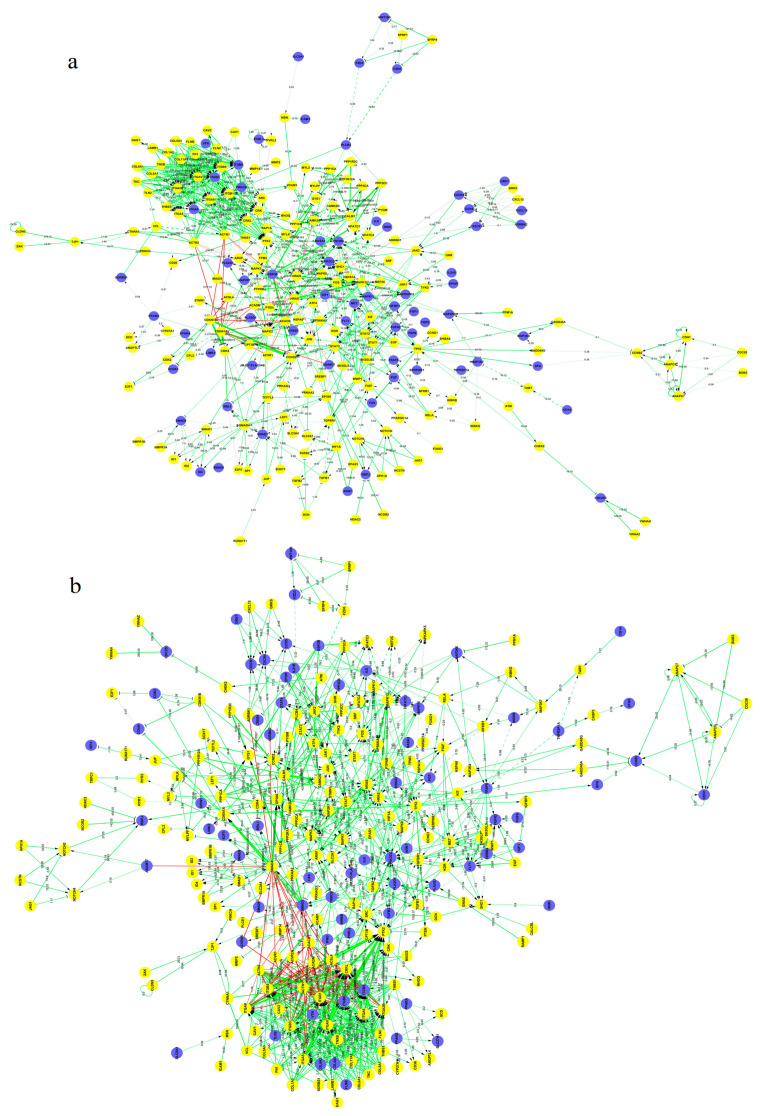
The interaction network of differentially expressed genes (DEGs) regulating myogenesis in Landrace (LR) (**a**) and Lantang (LT) (**b**) pig breeds. Yellow and blue dots represent DE and non-DE genes, respectively. Straight lines represent interaction association between genes. Solid and dashed lines represent direct and indirect interaction, respectively. Value and diameter of lines represent the interaction size [138].

Gene expression changes dramatically throughout the pig life cycle, particularly during prenatal growth and in the first stages of postnatal growth [138,139]. For example, *ACTC1*, *DLK1*, *COMP*, *ARHGAP36*, *FGF21*, *TNN*, *IBSP*, and *ATP6V0D2*, which are related to the development of various tissues, including muscle, are markedly upregulated in newborn piglets [27]. In addition, the *RETN* gene, which is associated with lipogenesis and thus meat quality, was found to be upregulated at birth [27]. Ayuso et al. [27] identified several candidate genes that might regulate muscle growth and development in IB and IB × DU pigs (*MEFs*, *SIM1*, *TCF7L2* or *FOXO1*, *KLF1* or *IRF2*, *MYH10*, *PVALB*, *CTNNB*, *MYOG*, and *SOX4*). Comparison of the muscle transcriptomes of piglet [140] and weanling [141] IB and IB × DU pigs revealed that *MYH10* is upregulated in IB × DU pigs, proving its association with muscle development. Ayuso et al. [27] also identified candidate genes associated with protein degradation and turnover and thus related to muscle development (*CTSF*, *ADAMTS8* or *CELA2*, *HSPS1*, *HSPA4L*, *ELANE*, *MMP9*, *FBXO32*, and *DNAJA1*). Among 83 genes found to be upregulated in LDM in their research, *ZIC1* and *MMP13* control myogenesis and myostatin signaling [142,143,144,145]. Using Solexa/Illumina’s genome analyzer in Lantang (LT, obese) and Landrace (LR, lean) pig breeds, Zhao et al. [138] identified 595 differentially expressed myogenesis genes (Figure 4). Moreover, they identified that *IKBKB, ACVR1, GSK3B, STMN1,* and *ITGA* might cause more muscle fibers in LR compared to LT. In comparison with LR, LT showed higher expression of several important inhibitors of myogenesis including *HMOX1, NOTCH2, CTNNA1, ID1, ID2, SMAD4, GPC3, MSTN*, and *CABIN1*, which might lead to slowing down the process of muscle differentiation in LT than in LR.

**Figure 5:**
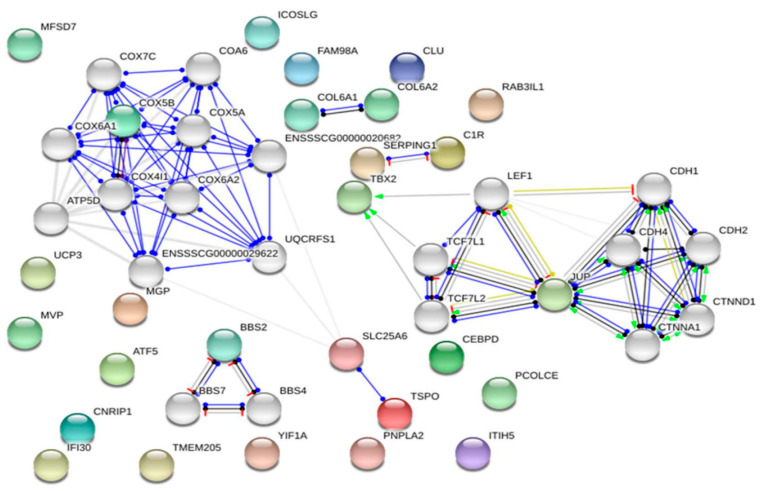
Upregulation of differentially expressed genes in the muscle of pigs with greater-than-normal muscle mass. Identified genes were colored and marked in grey; line shape represents anticipated mode of action. Lines colored represent anticipated mode of action (red shows interactions that were experimentally identified; blue shows interactions from selected databases; black shows coexpression; green shows text-mining relations and interactions contingent on appropriate researches stating a transfer from different organisms; and yellow shows transcriptional adjustment [164].

Genes related to an increased number of muscle fibers and the regulation of late myogenesis (*STMN1*, *ACVR1*, *GSK3B*, *IKBKB*, and *ITGA*) were identified in the Landrace breed [146]. In an RNA-sequencing analysis focusing on myogenesis phases in 40-, 55-, 63-, 70-, and 90-day Tongcheng and Yorkshire pig fetuses, Liu et al. [147] identified candidate genes associated with muscle growth and responsible for developmental differences between the breeds (*PPP1CC*, *EP300*, *MYO9A*, *PTEN*, *CDK14*, and *IRS1*). The *Akirin2* gene has been reported to be a promising candidate gene affecting porcine muscle satellite cell proliferation and differentiation, acting via the ERK1/2 and NFATc1 signaling pathways [148]. The *LEF1* gene, which is associated with the Wnt signaling pathway, plays an indispensable role in muscle fiber development by regulating the expression of MRFs such as *MYF5* and *MYOD*. Reis et al. [149] recognized *LEF1* as a promising candidate gene whose differential expression affects muscle mass; they observed greater expression of this gene in 3-week-old and 40-day-old fetuses of Duroc × Landrace × Large White cross pigs than in Piau pigs, associated with somite construction and proliferation and myoblast fusion. Notch genes (*Notch1–4*) have different expression patterns during prenatal and postnatal tissue development in mammals. *Notch1*, but not *Notch2* or *Notch3*, plays a key role in porcine satellite cell function, regulating the expression of the *HES5* gene, which controls *MYOD* and *MYOG* expression. *Notch1* also controls *GSK3β-3* gene expression, which has an important role in the Wnt signaling pathway [146,150]. Li et al. [151] examined the systematic correlation between obesity and DNA methylation and generated gene expression maps for muscle and adipose tissue. They identified favorable expression of *ACE*, *PRKAR1A*, *PRKCQ*, and *GHSR* in muscles; these genes stimulate the release of growth hormone and increase lean mass in obese pigs [152]. In Berkshire pigs, the hydroxysteroid 17-beta dehydrogenase 4 gene may influence muscle development, and it has been found to play a key role during early myogenesis, when its mRNA expression is substantially elevated [153]. Some genetic mutations have major impacts on muscle fiber number and size in pigs; many are related to muscle hypertrophy and in some situations affect meat quality [154]. A mutation in the *RYR1* gene causes lean muscle growth and muscle hypertrophy in heterozygous pigs [132]. Mutations in the *IGF2* and *PRKAG3* genes result in enhanced muscle mass in pigs [155]. Analysis of the LDM transcriptomes from Iberian pigs with divergent intramuscular fat content led to the identification of several candidate genes related to skeletal muscle development (*FOSB*, *FOS*, *CHAC1*, *ATF3*, *PPP1R15A*, *JUNB*, *DUSP1*, *PPP1R1B*, *NR4A2*, *CYRS1*, *EGR1*, *EGR2*, *EGR3*, and *POU3F1*) [156]. Moreover, Muñoz et al. [156] established 18 gene networks; network 1 contained many genes associated with skeletal and muscular system development (*ACTC1*, *ASB10*, *AHSP*, *ARHGEF4*, *ARHGEF6, DMD*, *JUNB*, *LMO7*, *MYL12A*, *RPRM*, *SDR16C5*, *SIRT3*, and *UCK2*). Analyses of regulatory impact factors led to the identification of genes associated with adipogenesis (*ARID5B*, *CREB1*, *VDR*, *ATF6*, and *SP1*) and myogenesis (*KLF11* and *MYF6*) [156]. In an analysis of the pig LDM transcriptome, Lobjois et al. [157] identified candidate genes in different functional networks: cell proliferation and myogenic differentiation (*AK1*, *BAG3*, *BNIP3*, *CDC34*, *CKM*, *COL1A2*, *CSNK2B*, *DES*, *FKBP8*, *GSN*, *HSP90AA1*, *IGF2*, *SET*, *TNNC2*, and *TNNT3*), and energy metabolism and muscle development (*COX8*, *DYSF*, *EEF2*, *IPO13*, *MYOZ1*, *NEB*, *TPM1*, *TTN*, *H19*, *NOP17*, and *RPL13A*). *MYH7*, *MYL1*, and *MYL3*, associated with muscle development, were also identified via transcriptome analysis in pig. Using RNA sequencing in combination with other livestock genomics research, Guo et al. [158] mentioned *SFRP2*, *KDM6A*, and *OGT* as candidate genes associated with pig growth traits. *SFRP2* has been shown to play key roles in muscle development and muscle satellite cell proliferation and differentiation in mice [159,160], and to affect skeletal muscle development during embryogenesis in pigs [158]. RNA sequencing led to the identification of *ASB2*, *MSTN*, *ANKRD1*, and *ANKRD2* as functional candidate genes associated with skeletal muscle development in Wei and Yorkshire pigs [161]. *MSTN* expression was found to be lesser in Wei pigs than in Yorkshire pigs, suggesting that this gene inhibits muscle growth less in the former breed [161]. *ASB2*, which has a negative regulatory effect on muscle mass [162], was also found to not be conductive to muscle growth in Wei pigs [161]. *ANKRD1* and *ANKRD2* belong to the muscle ankyrin repeat protein family and are known to regulate skeletal muscle cell differentiation [163]. To elucidate the genetic background affecting meat content, Ropka-Molik et al. [164] used microRNA expression profiles to identify DEGs that were upregulated and downregulated in pigs with greater muscle mass. They identified the *SOX2*, *SIRT1*, *KLF4*, *PAX6*, and transforming growth factor-beta genes as candidates likely associated with muscle mass, and found that *SETD2*, which encodes a histone methyltransferase, had modified transcript levels determined by loin mass. This gene is associated with epigenetic mechanisms controlling myoblast proliferation and differentiation, and its silencing results in cell-cycle arrest [165]. Taken together, the functions of this gene are crucial for muscle development and growth in pigs. In addition, the *TBX2* gene, which encodes the T-box transcription factor, is upregulated in pigs with greater muscle mass (Figure 5). *TBX2* controls the cell cycle in skeletal muscle through interaction with *MYOD* and cell-cycle regulators, including *p21* and *p14* [166].

## 8. Candidate Genes Affecting Muscle Development in Chickens

The consistently increasing demand for poultry meat has stimulated breeding of chickens with greater production value [167]. Controlled selection is performed to enhance the growth rate, feed efficiency, and carcass meat content of chicken [168]. Chicken muscularity and muscle growth are economically important characteristics with substantial agricultural implications. Thus, selection for chickens with increased muscle mass and acceptable meat quality is important to achieve consumer satisfaction. An understanding of the mechanisms underlying muscle development and growth and the identification of related candidate genes are key for efficient selection for poultry production.

Chickens bred for meat (broilers) and egg production (layers) are used in the best-known models for the investigation of the mechanisms underlying the regulation of the myogenesis rate and muscle development. In past decades, selection has focused primarily on the improvement of the broiler growth rate and muscle mass. Zheng et al. [169] examined gene expression differences in muscle between layers and broilers by microarray hybridization, and identified several genes associated positively or negatively with growth rates (and thus likely involved in muscle growth and development). They determined that the slow growth rate of layers relative to that of broilers was related to the higher expression levels of slow-type muscle-related genes (*MB*, *MYH7B*, *TNNI1*, *MYL3*, and *MYL2B*) and identified DEGs associated with satellite cell proliferation and differentiation, as well as muscle hypertrophy (*MUSTN1*, *FHL2*, *FGFR2*, *HS6ST2*, and *CSRP3*; Table 1). *CSRP3* was reported to be downregulated in humans with pathogenic cardiac hypertrophy [170]. *FHL2* expression in muscle precursor cells stimulated myoblast differentiation in mice [171], and it may control muscle growth and hypertrophy via the regulation of satellite cell proliferation and differentiation in chickens. The expression level of *MUSTN1*, a candidate gene related to muscle development that is upregulated after exercise in hypertrophic muscle [172], was greater in broilers than in layers, suggesting that this gene is a key regulator of skeletal muscle hypertrophy [169]. Broiler chickens also have lower expression levels of *FBXO22*, *FBXO30*, *UCHL1*, *RNF12*, *HERC4*, *RLD5*, and *HERC5* than do layers; these genes are associated with protein degradation and may be responsible for the greater muscle mass of broilers compared with layers [169]. Zheng et al. [169] also reported that *DKK3* may be associated with muscle development in chickens, as it is expressed more in broilers than in layers, in accordance with the divergent muscle growth rates of the two chicken lines. *DKK3* is known to suppress tumor growth by inhibiting cancer cell proliferation in human [173,174,175]. In another study, *POMC*, *NMU*, *NPW*, *PMCH*, *GAL*, and *FOS* were determined to be related to the broiler growth rate, as variable growth was attributed to their differential expression in the hypothalamus [176]. In a comprehensive study of DEGs that included principal component analysis, Nihashi et al. [177] identified 13 candidate genes potentially related to myoblast proliferation and differentiation in chickens (*CDKN2B*, *ACTC1*, *MYH15*, *TNNI1*, *TNNI2*, *TNNT2*, *CCK*, *CXCL14*, *MDK*, *PENK*, *CSRP2*, *MFAP5*, and *UCHL1*). These genes play crucial roles in muscle cell differentiation in chickens. *CXCL14* knockdown stimulates myogenic differentiation through the suppression of the myoblast cell cycle in mice [178]. *CSRP3* induces myoblast differentiation [179] and *CSRP2* is associated with the differentiation of smooth muscle cells in chicken [180]. The *CSRP2* expression level was high across differentiation in WL and UKC myogenic cells in chickens [177]. *MFAP5* tended to be more upregulated in WL than in UKC myogenic cells, indicating that it inhibits myotube formation in chickens. The level of *BMP4* expression in the hindlimbs of UKC broilers and WL layers was found to be highest at Hamburger–Hamilton (HH) stage 31–34 (E7–8) [181], suggesting that enhanced *BMP4* expression has a breed-dependent effect on myofiber formation. Using RNA sequencing, Xue et al. [182] analyzed the transcriptomes of Jinghai Yellow chicken muscle tissues in different early developmental phases. Their functional analyses resulted in the identification of DEGs related mainly to muscle development and growth (*GH*, *IGF2BP2*, *IGFBP3*, *CEBPB*, *FGF2*, and *IGFBP5*); in total, 44 candidate genes (including *FN1*, *MYH10*, *MAPK9*, *FGF2*, *CFL2*, *IRS1*, *PHKA1*, *FGF16*, *PHKG1* and *PHKB*) were related to differences in growth among developmental stages. They also identified genes associated with muscle differentiation and development (*MYOD1*, *MYLK2*, *SMYD1*, *BTG2*, *ANKRD2*, *PPP3CA*, *GPX1*, *TCF15*, *KLHL40*, and *CRYAB*). *CEBPB* is known to suppress myogenesis and activate adipogenesis [183,184] and to control several genes in response to GH [185].

GWASs have been used to identify gene loci and genomic regions related to muscle development more robustly and consistently than other approaches [186]. A GWAS led to the identification of five SNPs in 1.5 Mb of the *KPNA3-FOXO1A* region on GGA1 as related to muscle growth in an F2 resource chicken population [187]. Two of these SNPs were determined to affect the ADG, and the gene closest to them was recognized as *FOXO1A* [187], a crucial transcription factor for skeletal muscle development [188,189,190]. Several SNPs on GGA4 have been related significantly to chicken growth [191,192], and genes located close to them, such as LIM domain-binding 2, ligand-dependent nuclear receptor corepressor-like protein 1, and microtubule-associated protein tau, are less upregulated in the breast muscle of slow-growing relative to fast-growing chickens, suggesting that they are associated with muscle development [192]. Another GWAS led to the recognition that a region on GGA3 was related to the breast muscle percentage and weight in chickens [193]. The *GJA1* gene, located in this region, was identified as a candidate gene affecting muscle development, and its upregulation was found to be associated with increased breast muscle weight during development [193]. Several myogenic gene polymorphisms significantly related to muscle development (e.g., in *IGF1R* [194], *IGFBP2* [195], *IGF1* [196], *GHR* [197], *PIT1* [198], *GHSR* [199], *GHRL* [200], *MSTN* [201], *MEF2A* [202], *MYF5* [203], and *PAX7* [204]) have been identified in chickens. We also identified polymorphism of *UCP* gene associated with growth traits in Mazandaran indigenous chicken [205]. *UCP2* gene is expressed hugely in several tissues such as adipose and skeletal muscle in human [206]. Although there is a lack of information regarding the association of *UCP* gene and muscle development in chicken, we hypothesized that this gene has potential to be associated with muscle growth in chicken. 

Using high-throughput sequencing and microarray experiments, scientists have identified numerous candidate genes associated with skeletal muscle development in chickens. RNA sequencing of skeletal muscle samples from white rock and Xinghua chickens revealed greater expression of *FOXO3*, which may suppress skeletal muscle development in the Xinghua breed; decreased *FOXO3* expression may upregulate growth-related genes in DF-1 cells [207]. A comparison of DEGs between embryonic day 14 and week 7 in normal and sex-linked dwarf chickens led to the identification of numerous genes involved in the Wnt, insulin, MAPK, and PI3K-Akt pathways and associated with chicken muscle development; the interaction of these genes with miRNAs leads to the formation of regulatory (e.g., miRNA–mRNA) networks for skeletal muscle development [208].

Several miRNAs have been reported to have regulatory effects in different stages of muscle development. Integrative miRNA target prediction and network analysis led to the identification of many candidate genes and miRNA targets related to skeletal muscle development in chickens; for example, *gga-miR-1a* inhibits *ACVR2B* gene expression, and *RECK* is the target of *gga-miR-200b* [169]. In another study, *let-7b* was found to inhibit *GHR* gene expression and thus to play an important role in skeletal muscle development in chickens [209]. The *miR-206* gene was also found to play a critical role in chicken muscle development, with a proven effect on chicken birth weight [210]. In vitro experiments showed that *miR-203* inhibits myoblast proliferation and differentiation in chickens through the suppression of *c-JUN* and *MEF2C*, respectively [211]. *c-JUN* and *E2F1* play crucial roles in the stimulation of myoblast proliferation in chickens via the control of their target genes [211,212].

## 9. Conclusions

Muscle development is a complex but consistent process that can be improved substantially in farm animals through selection and be further enhanced by identification of related candidate genes. During the past years, significant progress has been made for understanding the mechanisms of muscle growth and development, and thus, we decided to write a comprehensive review covering regulators and (candidate) genes crucial for muscle development and growth in farm animals. We have highlighted important regulators including transcription factors, myogenic kinome, and GFs, and investigated the role of them on different aspects of muscle growth. Moreover, the role of many important (candidate) genes such as *MTPN*, *IQUB*, *NDUFA5*, *ASB15*, *LMOD2*, and *WASL* (beef cattle), differentially methylated genes (sheep, chicken), and the interaction network of DEGs (pig) on muscle growth in farm animals were discussed. Identification of such important regulators and genes offers enormous help for marker-assisted selection, plays an important role toward the goal of increasing meat yields, and helps breeders to maximize meat quantity and quality. In addition, the gene sets mentioned have the potential to be generally beneficial to the applied study of mammalian muscle growth. However, the mechanisms underlying muscle development and growth in farm animals especially beef and sheep need further explorations. 

## Figures and Tables

**Table 1:** Genes associated with muscle growth in broiler and layer chickens.

Gene Name	Symbol	Fold Change *				
		1D	2W	4W	6W	8W
**Slow-type myofiber protein genes**						
Troponin I type 1 (skeletal, slow)	*TNNI1*	−1.99	1.05	−6.47	−1.05	1.14
Myoglobin	*MB*	1.04	−1.81	−10.04	−6.8	1.17
Myosin, light chain 3, alkali; ventricular, skeletal, slow	*MYL3*	−1.96	−1.04	−19.79	−1.41	−1.02
Myosin, heavy chain 7B, cardiac muscle, beta	*MYH7B*	−3.07	−1.01	−11.31	1.1	1.36
Similar to myosin L2B regulatory light chain, cardiac muscle – chicken	*LOC417506*	−1.27	1.04	−58.02	−5.54	1.34
Myelin basic protein	*MBP*	1.16	−1.81	−1.83	−3.67	−1.68
Similar to dynein light chain-2	*LOC417663*	−1.25	−2.13	−1.77	−1.28	−1.73
**Satellite cell proliferation and muscle hypertrophy**						
Cysteine and glycine-rich protein 3 (cardiac LIM protein)	*CSRP3*	−2.17	1.01	−9.44	−16.72	−1.04
Four and a half LIM domains 2	*FHL2*	−1.38	−8.19	−10.95	−7.65	−9.84
Similar to actin binding LIM protein family member 2	*LOC422866*	−1.08	−2.06	−1.78	−2.24	−2.56
Fibroblast growth factor receptor 2	*FGFR2*	−1.31	−2.28	−1.05	−1.6	−1.87
Fibroblast growth factor 1 (acidic)	*FGF1*	−1.11	−2.45	−2.07	−2.75	1.61
Heparan sulfate 6-O-sulfotransferase 2	*HS6ST2*	−3.07	−4.09	−1.51	−2.13	−3.1
Musculoskeletal, embryonic nuclear protein 1	*MUSTN1*	−1.01	2.94	2.17	2.45	1.51
Fibroblast growth factor 16	*FGF16*	−1.06	2.86	2.45	8.16	2.77
Inner centromere protein antigens 135/155 kDa	*INCENP*	2.02	1.59	2.15	1.39	1.3
NudE nuclear distribution gene E homolog 1 (A. nidulans)	*NDE1*	1.49	2.48	2.68	1.91	1.23

* Comparison of expression values between muscle cells of broilers and layers, which normalized to a fold change of 1.0 and −1.0 in case of broilers having more and less mRNAs, respectively. D stands for day and W stands for week [169].

## Data Availability

Not applicable.

## Abbreviations

The following abbreviations are used in this manuscript: MSC: mesenchymal stem cell; MRF: myogenic regulatory factor; MEF2: myocyte enhancer factor 2; miR-1: microRNA-1; LWS: low-weight-select; HWS: high-weight-select; qRT-PCR: quantitative real-time reverse-transcription polymerase chain reaction; LDM: longissimus dorsi muscle; PKA: protein kinase; Rb: retinoblastoma protein; E2F: E2 factor; ERK: extracellular signal-regulated kinase; FGF: fibroblast growth factor; IGF: insulin-like growth factor; mTOR: mammalian target of rapamycin; HGF: hepatocyte growth factor; TGF: transforming growth factor; BTA: bovine chromosome; DM: double-muscled; QTL: quantitative trait loci; GWAS: genome-wide association studies; CAST: calpastatin; SHISA 9: Shisa family member 9; MSL1: complex subunit 1; CDK: cyclin-dependent kinases; SNP: Single nucleotide polymorphism; NGS: Next-generation sequencing; MTPN: Myotrophin; GDF-8: myostatin; EMA: eye muscle area; ADG: average daily gain; OAR: ovine chromosome; MYLPF: myosin light chain phosphorylatable fast skeletal muscle; MYH: myosin heavy chains; TPM2: tropomyosin 2; TTN: Titin; LT: Lantang; LR: Landrace; DEG: differentially expressed genes; HH: Hamburger–Hamilton; TNNI1: troponin I; MB: myoglobin; MYL3: myosin, light chain 3; MBP: myelin basic protein; CSRP3: cysteine and glycine-rich protein 3; FHL2: four and a half LIM domains 2; HS6ST2: heparan sulfate 6-O-sulfotransferase 2; MUSTN1: musculoskeletal, embryonic nuclear protein 1; FGF16: fibroblast growth factor 16; INCENP: inner centromere protein antigens 135/155 kDa; and NDE1: NudE nuclear distribution gene E homolog 1 (A. nidulans).
